# Aging mechanisms and interventions that impact senior health: Introduction to a special issue on Geroscience

**DOI:** 10.1002/agm2.12086

**Published:** 2019-09-20

**Authors:** Sean X. Leng, Baohua Liu, Brian K. Kennedy

**Affiliations:** ^1^ Division of Geriatric Medicine and Gerontology Johns Hopkins University School of Medicine Baltimore MD USA; ^2^ Guangdong Key Laboratory of Genome Stability and Human Disease Prevention Department of Biochemistry & Molecular Biology School of Basic Medical Sciences Shenzhen University Shenzhen China; ^3^ Departments of Biochemistry and Physiology Yong Loo Lin School of Medicine Centre for Healthy Ageing National University of Singapore National University Health System Singapore City Singapore; ^4^ Singapore Institute for Clinical Sciences Agency for Science, Technology and Research (A*STAR) Singapore City Singapore; ^5^ Buck Institute for Research on Aging Novato CA USA

It is with distinct pleasure to introduce this September issue of Aging Medicine as a special issue dedicated to the International Geroscience Symposium entitled “Aging Mechanisms and Interventions that Impact Senior Health” held on May 24‐25, 2019 in Shenzhen, China. The Symposium was the first in a series of seven international geroscience conferences bringing together researchers from around the world to advance the revolutionary approach to healthy aging known as geroscience. It was held in conjunction with the 2019 annual scientific meeting of Chinese Medical Association Geriatrics Branch and China National Center of Geriatrics and Gerontology. The Symposium provided a truly unique and important international platform for promoting geroscience and brainstorming on its further development in China, the country with the world's largest aging population.

Aging itself alone, by far, is the greatest risk factor for almost all chronic conditions including frailty and disability in older adults. Geroscience aims to gain in‐depth understanding of biology and mechanisms of with the hypothesis that therapeutically addressing aging and its mechanisms directly will simultaneously prevent the onset or mitigate the severity of multiple chronic diseases (Figure 1, 1). This is innovative and much more efficient that the traditional approach of addressing one disease at a time. It also helps promote healthspan with profound medical, policy and socioeconomic impact.^2^ In 2011, the US National Institutes of Health (NIH) launched the Trans‐NIH Geroscience Interest Group (GSIG), which was initiated and led by the National Institute on Aging (NIA) with participation of 22 NIH institutes and centers.^3^ GSIG has since become a major driving force for advancing basic and translational aging research in the US. Among other symposia and conferences, GSIG has launched two major summits in the US, one entitled “Advances in geroscience: impact on healthspan and chronic disease” in 2013 and another entitled “Disease drivers of aging” in 2016 with high profile scholarly exchanges and research presentations as well as high impact scientific publications.^1^, ^4^ NIH has also provided funding to promote geroscience research (RFA AG‐16‐020, 3). Moreover, GSIG has been established at the American Geriatrics Society (AGS) and Gerontological Society of America (GSA) to further promote geroscience in geriatrics and aging research community in the US. Under the leadership of Dr. Felipe Sierra, Director of Aging Biology Program and his colleagues at NIA as well as a number of prominent extramural geroscience researchers, a series of seven international symposia were planned in 2019 across five continents to promote geroscience research globally. Such an international conference series is built upon the geroscience development momentum in the US and planned in collaboration with the American Federation for Aging Research with some funding from NIA's Nathan Shock Centers of Excellence. For this inaugural geroscience symposium in China, we were fortunate to secure additional funding from Milstein Medical Asian American Partnership (MMAAP) Foundation (http://www.mmaapf.org), a New York‐based not‐for‐profit foundation towards China and Greater Asia with its Irma and Paul Milstein Program for Senior Health dedicate to promoting the development of geriatric medicine and aging research.

**Figure 1:**
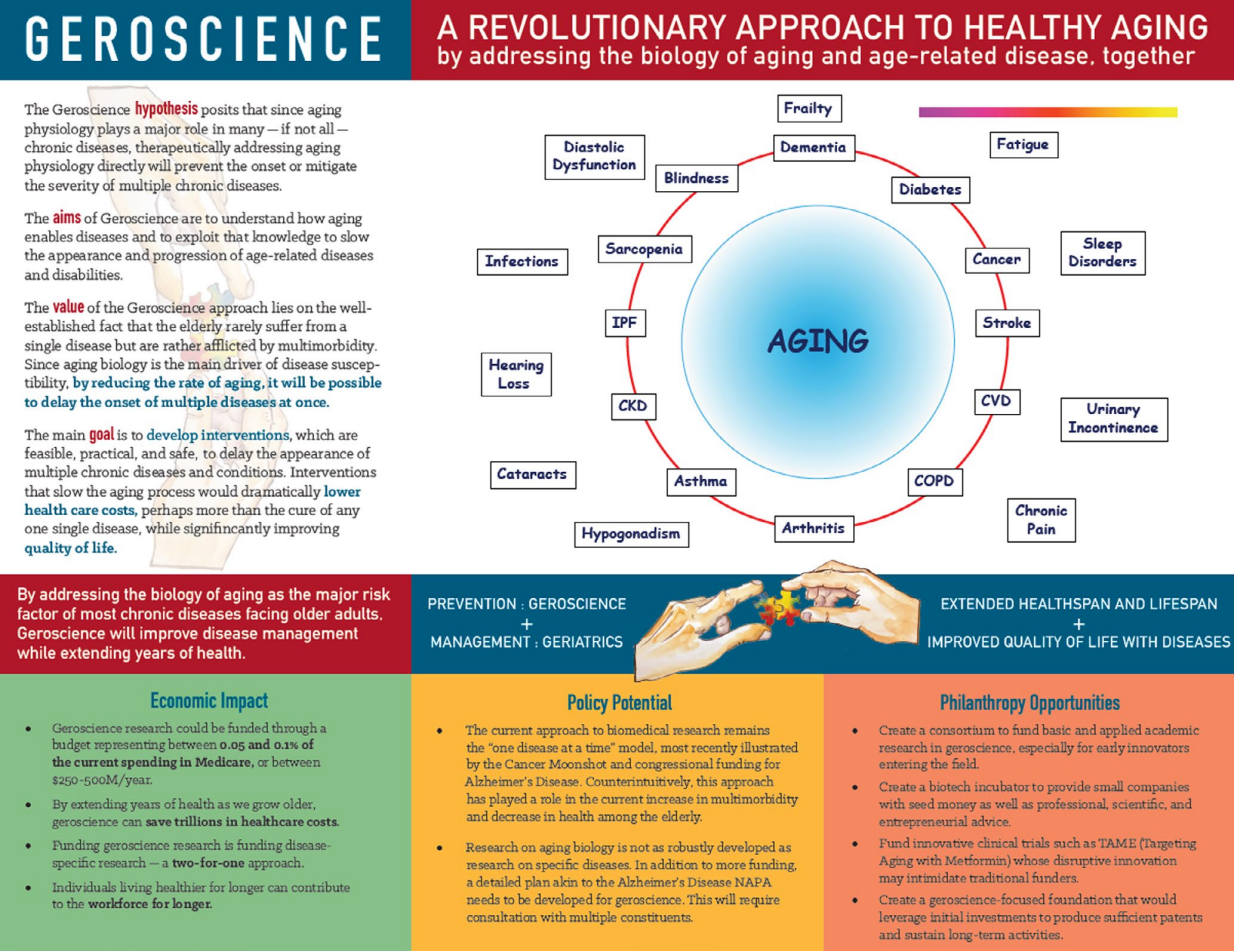
The conceptual framework of Geroscience: hypothesis and goals, linkage between aging and diseases, as well as its impact on senior health and society

As pointed out by Mr. Howard P. Milstein, Chairman of MMAAP Foundation, the decision of holding the first international geroscience symposium in China was significant, stating “Meeting in China—home to more older adults than any other nation on our planet—sends a powerful message to the global research community”.^5^ With the largest aging population in the world, the demographic shift in China is considered as a “tsunami”, whereas such shifts in many other countries are often referred to as “waves”. In China, the number of older adults (aged >60 years) reached 250 million at the end of 2018 and is projected to reach 487 million by 2053. In addition, those aged 80 and older reached 28 million at the end of 2018 and is projected to grow at a rate of one million per year. The Chinese government is acutely aware of this aging imperative. While the development of a geriatrics workforce and clinical services is emphasized, including a number of national policies issued by the central Chinese government on senior healthcare and support 6, 7 government investment in geroscience has also been impressive. For example, the National Health Commission (NHC, previously known as National Health and Family Planning Commission or Ministry of Health) funds Centers in Geriatrics and Gerontology. The initial cycle of funding was issued in 2015 to 32 large academic medical centers across China with over 10 million Chinese Yuan each. In 2016, the NHC established the National Center on Geriatrics and Gerontology with over 400 million Chinese Yuan and land, as well as a mandate on basic aging research. This is only the 3rd national center, after one for cancer and one for cardiovascular diseases, demonstrating the emphasis of government investment in geroscience. In addition, the Ministry of Science and Technology (MOST) has recently listed aging research as one of its funding priorities.^8^ In 2017, MOST funded six research centers with tens of millions of Chinese Yuan each across the country. These are just a few examples of government investment in geroscience at the national level. Provincial and local government agencies are developing numerous supportive policies and new initiatives for geriatrics and geroscience research as well. If this trend in funding continues, it is not hard to imagine China as a leader in geroscience research in the near future. At this symposium, there was a dedicated session on infrastructure and platform building for geroscience, key points from which are summarized in the articled by Liu, et al in this issue.

With much of the planning done by the authors of this article who are the co‐Chairs of the Symposium and other key thought leaders in geroscience as well as in collaboration with AFAR and leadership of Chinese Medical Association Geriatrics Branch and China National Center on Geriatrics and Gerontology, this two‐day Symposium was organized into seven sessions with each covering one of the following seven key topics of gerosceince research: (a) Principles of geroscience; (b) Pillars of aging, resilience & immune aging; (c) Anti‐aging research & translation; (d) Traditional Chinese Medicine & aging; (e) Stem cell research & regenerative medicine; (f) Artificial intelligence & aging; and (g) Infrastructure & platform building for geroscience. It featured more than 40 expert speakers from China, other Southeast Asian and European countries, as well as the United States (Figure 2A) with several hundreds of participants from academia (postdoctoral fellows, young and established clinicians and investigators in geriatric medicine and aging research, etc.), government (policy makers), NGOs, and industry (Figure 2B). Presentations by each of the expert speakers at the symposium were structured in a way that speakers provided the audience with a high level view of key facts and conceptual issues as they related to their research area rather than sharing latest research findings as it would be the case at a Gordon, Keystone, or other research conferences. To promote active discussion and brain storming on these key geroscience topics, at least 45 minutes were allocated at the end of each scientific session as well as the entire eighth/last session of 2 hours for discussion. In this special issue of Aging Medicine, we are pleased to present several articles on some of the key topics that are based on what was discussed in their respective sessions at the symposium, including (a) Geroscience and the challenges of aging societies contributed by Dr. Felipe Sierra; (b) Meta‐inflammaging at the crossroad of geroscience contributed by Drs. Guobing Chen and Raymond Yung; (c) Traditional Chinese medicine and geroscience: integration and collaboration promote healthy aging contributed by Drs. Weihong Cong and Keji Chen; (d) Osteosarcopenia: a case of geroscience contributed by Drs. Ben Kirk, Ahmed Al Saedi, and Gustavo Duque; (e) Allogeneic mesenchymal stem cell therapy: A regenerative medicine approach to geroscience contributed by Drs. Anthony Oliva and Joshua Hare; and (f) Geroscience infrastructure building in China contributed by Drs. Xiang Liu, et al. In addition, key points from the Discussion session towards the end of the symposium are summarized and presented here by Drs. Yiyin Chen and Sean X. Leng. This constellation of seven outstanding articles provide readers an excellent overview of take‐home points from the engaging discussions on respective topics at the Symposium. It is hoped that such exemplary work will facilitate further development of geroscience in China and around the world. We wish subsequent international geroscience conferences of this series great successes as well!

**Figure 2:**
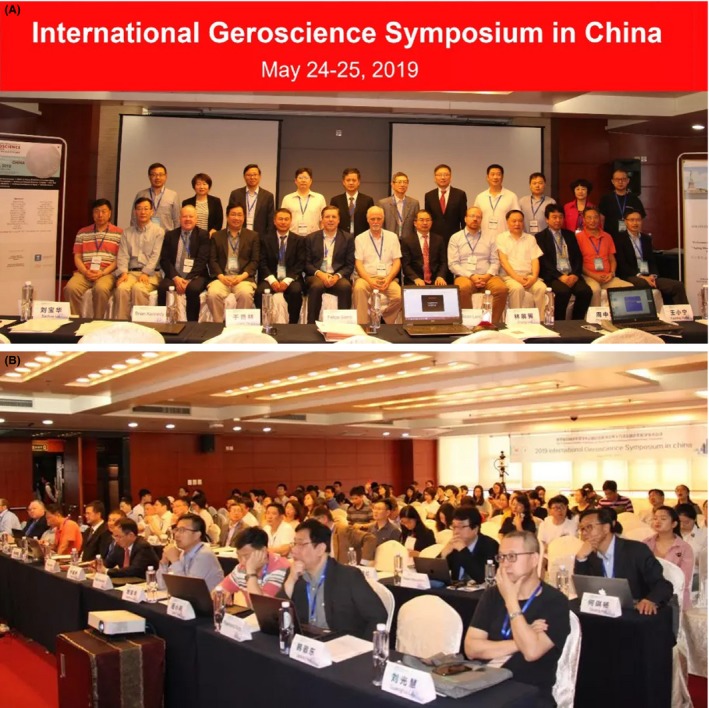
Photos of expert speakers (not all present, panel A) and audience (panel B) at the International Geroscience Symposium in Shenzhen, China. May 24, 2019

## CONFLICTS OF INTEREST

S.X.L. is President of the Milstein Medical Asian and American Partnership Foundation and B.K.K. is on the grant review panel. B.K.K. is Director of the Centre for Healthy Ageing in the National University Health System in Singapore. B.K.K. is a Scientific Advisor and on the Board of Directors at PDL Pharma, is on the Board of Directors at L‐Nutra, is on the Board of Directors at Mt. Tam Biotechnologies, and performs corporate sponsored research for Gero LLC.
